# Antigen spreading-induced CD8^+^T cells confer protection against the lethal challenge of wild-type malignant mesothelioma by eliminating myeloid-derived suppressor cells

**DOI:** 10.18632/oncotarget.5856

**Published:** 2015-09-28

**Authors:** Zhe Yu, Zhiwu Tan, Boon Kiat Lee, Jiansong Tang, Xilin Wu, Ka-Wai Cheung, Nathan Tin Lok Lo, Kwan Man, Li Liu, Zhiwei Chen

**Affiliations:** ^1^ AIDS Institute and Department of Microbiology, Li Ka Shing (LKS) Faculty of Medicine, The University of Hong Kong, Hong Kong SAR, P.R. China; ^2^ Department of Orthopedic Surgery, Orthopedics Oncology Institute of Chinese PLA, Tangdu Hospital, Fourth Military Medical University, Xi'an, Shaanxi, P.R. China; ^3^ Department of Surgery and Centre for Cancer Research, LKS Faculty of Medicine, The University of Hong Kong, Hong Kong SAR, P.R. China; ^4^ Research Center for Infection and Immunity, LKS Faculty of Medicine, The University of Hong Kong, Hong Kong SAR, P.R. China

**Keywords:** CD8+T cells, vaccination, mesothelioma, antigen spreading, MDSCs, Immunology and Microbiology Section, Immune response, Immunity

## Abstract

A key focus in cancer immunotherapy is to investigate the mechanism of efficacious vaccine responses. Using HIV-1 GAG-p24 in a model PD1-based DNA vaccine, we recently reported that vaccine-elicited CD8^+^ T cells conferred complete prevention and therapeutic cure of AB1-GAG malignant mesothelioma in immunocompetent BALB/c mice. Here, we further investigated the efficacy and correlation of protection on the model vaccine-mediated antigen spreading against wild-type AB1 (WT-AB1) mesothelioma. We found that this vaccine was able to protect mice completely from three consecutive lethal challenges of AB1-GAG mesothelioma. Through antigen spreading these animals also developed tumor-specific cytotoxic CD8^+^ T cells, but neither CD4^+^ T cells nor antibodies, rejecting WT-AB1 mesothelioma. A majority of these protected mice (90%) were also completely protected against the lethal WT-AB1 challenge. Adoptive cell transfer experiments further demonstrated that antigen spreading-induced CD8^+^ T cells conferred efficacious therapeutic effects against established WT-AB1 mesothelioma and prevented the increase of exhausted PD-1^+^ and Tim-3^+^ CD8^+^ T cells. A significant inverse correlation was found between the frequency of functional PD1^−^Tim3^−^ CD8^+^ T cells and that of MDSCs or tumor mass *in vivo*. Mechanistically, we found that WT-AB1 mesothelioma induced predominantly polymorphonuclear (PMN) MDSCs *in vivo*. In co-cultures with efficacious CD8^+^ T cells, a significant number of PMN-MDSCs underwent apoptosis in a dose-dependent way. Our findings indicate that efficacious CD8^+^ T cells capable of eliminating both tumor cells and MDSCs are likely necessary for fighting wild-type malignant mesothelioma.

## INTRODUCTION

Immune surveillance is considered to be an important host defense mechanism to maintain cellular homeostasis and to inhibit carcinogenesis [1]. Effector CD8^+^ T cell or cytotoxic T lymphocyte (CTL) plays a key role in immune surveillance by eliminating antigen-specific target cells during tumor immunotherapy [2]. The universality of MHC class I molecule expression allows CTLs to selectively recognize peptides from diverse tumor antigens [3]. After the recognition of peptide-MHC complex, CTLs can lyse target tumor cells with the involvement of perforin and/or granzymes, and by releasing cytokines (e.g., IFN-γ) and initiating death receptor-mediated apoptosis pathways [4, 5]. Therefore, the induction of high frequency of long-lasting effector CD8^+^ T cells has been a key focus in cancer immunotherapy research.

We previously reported that a PD1-based DNA vaccine, namely sPD1-p24_fc_, when delivered via *in vivo* electroporation (EP), induces a high frequency of antigen-specific CD8^+^ T cells with broad reactivity, long-term memory, polyfunctionality and cytotoxicity [6]. Furthermore, using this model sPD1-p24_fc_/EP vaccine, we recently demonstrated that vaccine-elicited CD8^+^ T cells conferred complete prevention and therapeutic cure of AB1-GAG malignant mesothelioma [5]. The efficacy was attributed to vaccine-elicited CD8^+^ T cells that could retain their effector functions once infiltrated into the tumor [7], reduce myeloid-derived suppressor cells (MDSCs) and CD4^+^CD25^+^Foxp3^+^ regulatory T lymphocytes (Treg) cell populations [8, 9], and lead to the complete clearance of tumor cells [5, 7]. Thus, if the vaccine is highly potent, it is possible to use active vaccination to harness the immune system and reinstate immune surveillance by overcoming tumor-associated immune suppression.

Currently, vaccine-based cancer immunotherapy remains largely hindered by the lack of potent tumor antigens and by the tumor-induced immune suppressive cells such as MDSCs [10]. For example, despite its immunogenic potential of wilms’ tumor protein 1 (WT1) in mice and clinical trials [11], our data indicated that a WT1-based vaccine was not able to induce potent CD8^+^ T cells to either prevent or cure WT1-expressing mesothelioma [5]. Thus, it becomes critical to investigate if there are any other mesothelioma antigens for eliciting efficacious CD8^+^ T cells. As for tumor-induced immune suppression, MDSCs originated from the bone marrow are largely accumulated in tumor microenvironments [12]. MDSCs are a phenotypically heterogeneous population consisting of monocytic MDSCs (M-MDSCs) and polymorphonuclear MDSCs (PMN-MDSCs), of which both can dampen the immune response through the inhibition of T cell activation and proliferation [9, 13]. Efficacious CD8^+^ T cells, therefore, should overcome the immune suppressive effects of tumor-induced MDSCs [5, 14].

Based on these observations and publications by others [15, 16], we hypothesized that antigen spreading after vaccine-induced CTL killing of AB1-GAG mesothelioma cells should be immunogenic for triggering tumor-specific immune responses against wild-type AB1 mesothelioma, namely WT-AB1.. We show here that antigen-spreading during the repeated eliminations of AB1-GAG mesothelioma by sPD1-p24_fc_/EP vaccinations indeed resulted in the generation of effective tumor-specific cytotoxic CD8^+^ T cells, which were capable of inhibiting PD1/Tim3 expression on their surface, reducing the number of MDSCs, and rejecting WT-AB1 malignant mesothelioma.

## RESULTS

### sPD1-p24_fc_/EP DNA vaccination protects mice completely against three consecutive lethal challenges of AB1-GAG malignant mesothelioma

**Figure 1:**
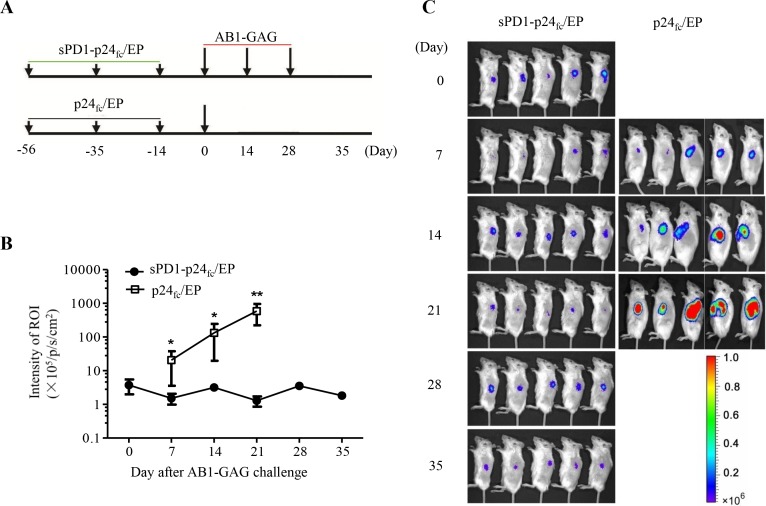
Complete protection of mice against three consecutive lethal challenges of AB1-GAG mesothelioma **A.** Schematic representation of a prophylactic study on the sPD1-p24_fc_/EP DNA vaccine against repeated AB1-GAG mesothelioma challenges. **B.** After three consecutive lethal challenges of 5 × 10^5^ AB1-GAG mesothelioma cells, tumor growth was assessed by BLI as compared with p24_fc_/EP-vaccinated mice. **P = 0.007. Data was plotted as mean ± s.e.m. **C.** Representative BLI of AB1-GAG tumor growth in vaccinated mice according to the experimental time schedule **A.**. Images were acquired at the indicated time points after tumor cell inoculation. The color scale indicates the BLI signal intensity. Two independent experiments generated the same results.

In a previous study, we demonstrated that high frequency of CD8^+^ T cells elicited from sPD1-p24_fc_/EP vaccination achieved complete and long-lasting protection of BALB/c mice from two lethal AB1-GAG challenges that expresses the same p24 antigen [5]. In order to develop a model for the induction of anti-tumor immune responses following in situ tumor destruction, we sought to increase the frequency of AB1-GAG challenge up to three times while shortening the time span of each implantation. By the same immunization protocol [6, 17], we vaccinated groups of BALB/c mice intramuscularly (i.m.) *via* immediate electroporation (EP) over the injection site three times at three-week intervals with 100 μg plasmid DNA of sPD1-p24_fc_, p24_fc_ or PBS control in a volume of 100 μl. Two weeks after the last immunization, three consecutive rounds of subcutaneous (s.c.) AB1-GAG inoculations were performed at two-week intervals on their left flank (Figure 1A). We consistently found that all sPD1-p24_fc_/EP vaccinated mice cleared implanted AB1-GAG cells within two weeks and survived after the consecutive tumor challenges (Figure 1B and 1C). In contrast, none of the animals in control groups could withstand one time tumor challenge and died within 4-6 weeks. Bioluminescence imaging (BLI) was taken every week after tumor implantation. Comparison was made based on the intensity of luciferase signal from the region of interest (ROI), showing that vaccination with sPD1-p24_fc_/EP led to a significant suppression of AB1-GAG tumor progression (Figure 1B and 1C, **P = 0.007). These results suggested that sPD1-p24_fc_/EP vaccination effectively eliminated three times of AB1-GAG malignant mesothelioma challenges, resulting in the establishment of a vaccine-mediated tumor destruction model. This model provided a useful system to address the critical question of whether three times of AB1-GAG elimination would induce antigen spreading and lead to the induction of tumor-specific immunity against WT-AB1.

### Mice protected from AB1-GAG challenges generate antibody and CD8^+^ T cell responses against WT-AB1 malignant mesothelioma

**Figure 2:**
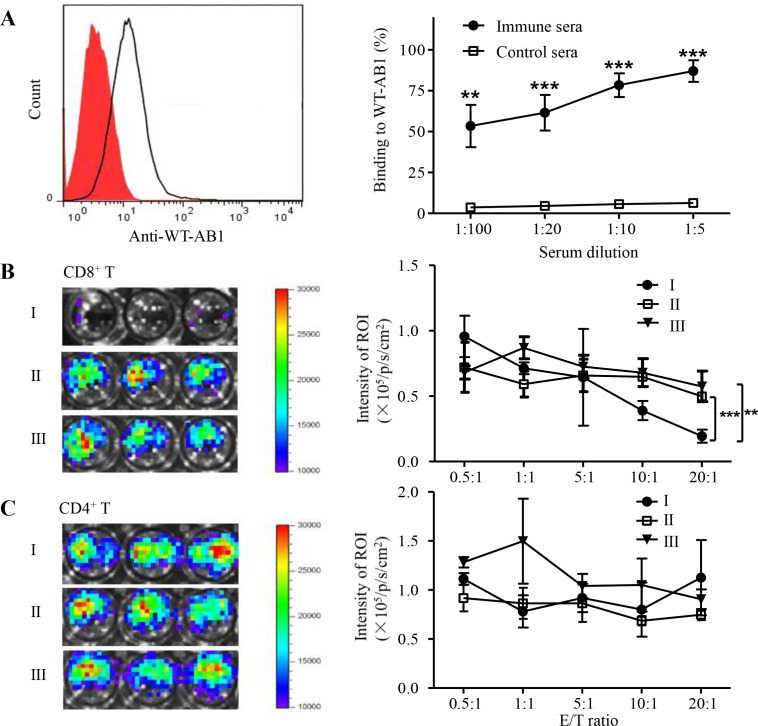
Tumor-specific immune responses after three consecutive lethal challenges of AB1-GAG mesothelioma **A.** Antibody binding to WT-AB1 cells by flow cytometry. Representative histogram shows the binding of 1:100 diluted anti-sera to WT-AB1 cells with the fluorescent signal shifted to the right (unfilled peak) as compared to control sera (red filled peak). Pre-labeled anti-mouse IgG was used as the detection antibody (left panel). Using various serum dilutions, immune sera show consistent binding to WT-AB1 cells in a dose-dependent way as compared with the control sera (right panel). **P < 0.01; ***P < 0.001. **B.** *In vitro* CD8^+^ T cell cytotoxic assay. Representative bioluminescence images depict the luminescence intensity in each well at the E/T ratio of 20:1 (left panel). The degree of CTL-mediated killing of tumor cells was indicated by the decrease of luminescence activity using the IVIS100 luminescence imaging system (right panel). BLI signals were acquired for 20 second. **P = 0.0066; ***P = 0.00078. **C.** *In vitro* CD4^+^ T cell cytotoxic assay. Representative bioluminescence images depict the luminescence intensity in each well at the E/T ratio of 20:1 (left panel). There were no CD4^+^ T cytotoxic activities against tumor cells because of the lack of significant differences between experimental groups (right panel).

Our previous data indicated that the presence of cytotoxic CD8^+^ T cells is an important factor responsible for the eradication of AB1-GAG by releasing inflammatory IFN-γ and TNF-α in the vicinity of target cells as well as by initiating TRAIL-directed tumor cell apoptosis [5]. Cell debris released after tumor destruction then becomes a potential antigen repertoire for reinitiating immune responses suppressed by the tumor microenvironment [18]. However, little is known regarding the functional characteristics of anti-tumor immune responses induced by the mesothelioma destruction. For this reason, we sought to determine antibody and T cell responses against WT-AB1 mesothelioma. Indeed, after three times of AB1-GAG elimination, we detected specific IgG antibodies that could react to WT-AB1 cells by flow cytometric analysis. As shown in Figure 2A, all protected mice have developed WT-AB1-specific antibody responses. The binding of anti-sera to WT-AB1 cells resulted in an increased fluorescent signal shifted dose-dependently to the right as compared to control sera (Figure 2A, left panel). Antibody-dependent cell mediated cytotoxicity (ADCC) may play a role in elimination of tumor cells through activating effector neutral killer (NK) cells [19]. Using the same method, we then measured the ADCC activities of these anti-sera derived from sPD1-p24_fc/EP_/AB1-GAG (group I), sPD1-p24_fc/EP_/PBS (group II) or placebo control mice (group III) at different dilutions (1:5, 1:10, 1:20 and 1:100). However, murine sera from three groups showed no significant difference (data not shown), thus, these reactive anti-sera, may not mediate ADCC effects against WT-AB1. Subsequently, we sought to measure T cell responses against WT-AB1. Both CD4^+^ and CD8^+^ T cells purified from splenocytes of immunized-protected mice were subjected to cytotoxicity assays, respectively. Luciferase-expressing WT-AB1 tumor cells (2 × 10^4^/well) were seeded as target cells in this experiment. Purified CD8^+^ or CD4^+^ T cells were assayed at different effector:target (E/T) ratio from 0.5:1 to 20:1. Clearly, CD8^+^ T cells isolated from group I mice showed a strong cytotoxic effect against WT-AB1 cells *in vitro* at E/T ratios of 10:1 and 20:1 (Figure 2B, ***P = 0.00078). In contrast, CD4^+^ T cells did not show significant cytotoxic activities (Figure 2C). Although both antibody and CD8^+^ T cell responses were elicited during the process of AB1-GAG elimination, only CD8^+^ T cells exhibited capacity in killing WT-AB1 cells. Therefore, under our experimental conditions cell debris released after AB1-GAG destruction likely induced tumor-specific CD8^+^ T cell immune responses against WT-AB1.

### Mice protected from AB1-GAG challenges show significant protection against WT-AB1 malignant mesothelioma

**Figure 3:**
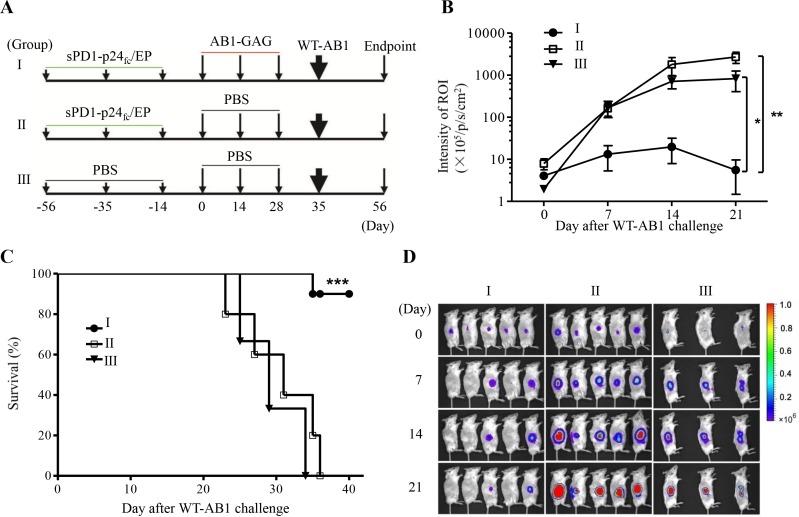
Protection of mice against the lethal challenge of wild-type mesothelioma **A.** Schematic representation of the study design. **B.** After a lethal dose of 5×10^5^ WT-AB1 challenge, tumor growth was assessed by BLI overtime. Statistical analysis was done on day 21 between groups I and II or III (*n* = 5 in I and II, 3 in III). Data was plotted as mean ± s.e.m. **P = 0.0098; *P = 0.0376. **C.** Survival curve. The majority of mice (90%, 9/10) in group I survived after the WT-AB1 challenge and remained tumor free. Results were combined from two independent experiments with *n* = 5 each. ***P = 0.0002. **D.** Representative BLI depicts the growth of WT-AB1 mesothelioma in one of two independent experiments. Images were acquired at the indicated time points after the inoculation of WT-AB1.

The induction of broad immune protection is highly desirable against tumors with unproven tumor antigens [20]. Cytolytic destruction of AB1-GAG tumor could lead to the modulation of antigen repertoire and spreading of tumor antigens that could induce tumor-specific T cell immunity responsible for tumor regression [21, 22]. Accordingly, we sought to determine whether these protected mice would also show prophylactic effects against WT-AB1 challenge. The challenge was performed with a lethal dose of 5 × 10^5^ WT-AB1 cells in the opposite right flank s.c. (Figure 3A). After the challenge, living images of tumor growth and animal survival were studied overtime (Figure 3B-3D). The sPD1-p24_fc/EP_/AB1-GAG (group I) proved to be significantly more efficient than controls at rejecting implanted WT-AB1 cells (Figure 3B, **P = 0.0098), and most protected mice (9/10, 90%) remained tumor-free. In contrast, all control mice developed tumors and died within 40 days (Figure 3C, ***P = 0.0002). As described in our recent study, although sPD1-p24_fc_/EP vaccination was fully protective against AB1-GAG challenges (Figure 1), it did not provide any protective benefits against the lethal challenge of WT-AB1 [5]. By way of explanation, the immune state achieved by three times of sPD1-p24_f_/EP vaccination did not confer any protection against WT1-AB1, which is also demonstrated through the results of (group II in this study (Figure 3). The observed anti-WT-AB1 protective immunity, therefore, must require specific anti-tumor immunity generated during the process of AB1-GAG elimination mediated by p24-specific CD8^+^ T cells.

### CD8^+^ T cell response is responsible for the protection of the lethal challenge of WT-AB1 malignant mesothelioma

**Figure 4:**
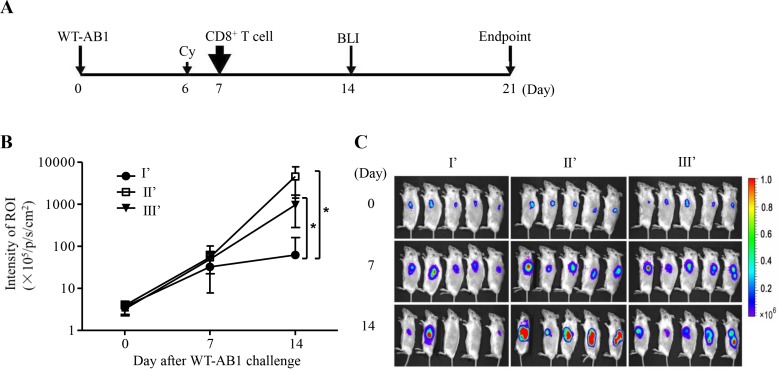
Adoptive transfer of tumor-specific CD8^+^ T cells eliminates established WT-AB1 malignant mesothelioma **A.** Time schedule of the adoptive transfer of CD8^+^ T cells. SCID mice were pre-inoculated s.c. with 5 × 10^5^ WT-AB1 cells, and tumors were left to grow for 7 days to reach approximately 5 mm in length. Cyclophosphamide (Cy) was injected intraperitoneally (150 mg/kg) one day before the adoptive transfer of CD8^+^ T cells. 2 × 10^6^ of isolated CD8^+^ T cells in 100 μl PBS were then injected *via* the tail vein into each mouse. **B.** Tumor growth was assessed by BLI overtime. CD8^+^ T cells of groups I, II and III were adoptively transferred i.v. into SCID mice of groups I’, II’ and III’, correspondingly. Intravenous transfer of CD8^+^ T cells of group I significantly eliminated established WT-AB1 tumor in SCID mice of group I’ (*n* = 5). Data represent mean ± s.e.m. *P = 0.0194. **C.** Representative BLI of WT-AB1 tumor growth in three groups of SCID mice. Images were acquired at the indicated time points after WT-AB1 tumor inoculation.

We sought to further determine the correlation of immune protection against WT-AB1 *in vivo*. Using previously described experimental protocols [5], we conducted adoptive transfer of pooled anti-sera and purified CD8^+^ T cells derived from group I and control groups, respectively. Anti-sera from protected or unprotected BALB/c mice were transferred i.p. into immunodeficient SCID mice 3 days before the lethal challenge of WT-AB1, then another 3 boosts of the same anti-sera were administered at day 1, 3, 5 after the tumor inoculation. In consistency with the lack of *in vitro* ADCC activities, results of BLI indicated that none of the infused anti-sera showed elimination of WT-AB1 tumor (data not shown). We then sought to determine the role of CD8^+^ T cells in tumor rejection *in vivo* by conducting T cell adoptive transfer experiments in the SCID mouse model. For this purpose, under a therapeutic setting, a lethal dose of 5 × 10^5^ WT-AB1 cells were inoculated into SCID mice of groups I’, II’ and III’ 7 days before they received intravenous injection of purified CD8^+^ T cells from groups I, II and III, correspondingly (Figure 4A). As compared to groups II’ and III’, mice in group I’ receiving CD8^+^ T cells from protected mice showed significant elimination of WT-AB1 tumor (Figure 4B and 4C, *P = 0.0194). Again, HIV-1 p24-specific CD8^+^ T cells induced by three times of sPD1-p24_fc_/EP vaccination did not confer any protection against WT-AB1 in group II’ (Figure 4), which is in agreement with previous findings (group II in Figure 3) [5]

### Tumor-specific CD8^+^ T cells function by suppressing PD1/Tim3 expression on their surface and by eliminating PMN-MDSCs

**Figure 5:**
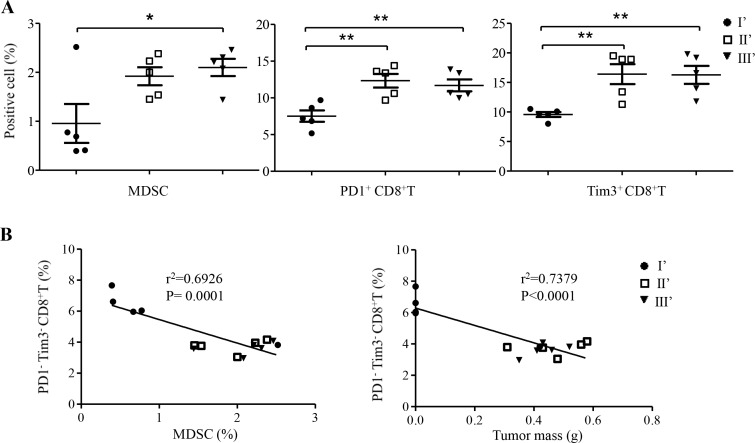
Frequency and correlation analysis of CD8^+^ T cells and MDSC in spleens of SCID mice of groups I’, II’ and III’ **A.** Frequencies of MDSC, PD1^+^ CD8^+^ T cells, and Tim3^+^ CD8^+^ T cells were measured at the experimental endpoint (Figure 4A). Data represent mean ± s.e.m. *P < 0.05; ** *P* < 0.01; ***P < 0.001. **B.** The frequency of PD1^−^Tim3^−^ CD8^+^ T cells was inversely correlated with that of MDSC (left panel) and with tumor mass (right panel), respectively.

To further illustrate the functionality of tumor-specific CD8^+^ T cells in modulating tumor-associated immunosuppression, we isolated splenocytes from protected and control SCID mice, and analyzed the frequency of MDSCs as well as the expression of exhaustion markers PD1 and Tim3 on CD8^+^ T cells. Tumor-free mice in group I’ consistently retained the low expression of PD1 (**P = 0.0058) and Tim3 (**P = 0.0028) on CD8^+^ T cells (Figure 5A) as well as significantly reduced frequencies of MDSC (*P = 0.0303). Interestingly, one unprotected mouse from group I’ displayed a high frequency of MDSCs. Correlation analysis indicated that the frequency of non-exhausted PD1^−^Tim3^−^ CD8^+^ T cells was inversely correlated with that of MDSC significantly (Figure 5B, left, ***P = 0.0001) as well as with tumor mass (Figure 5B, right, ***P < 0.0001).

**Figure 6:**
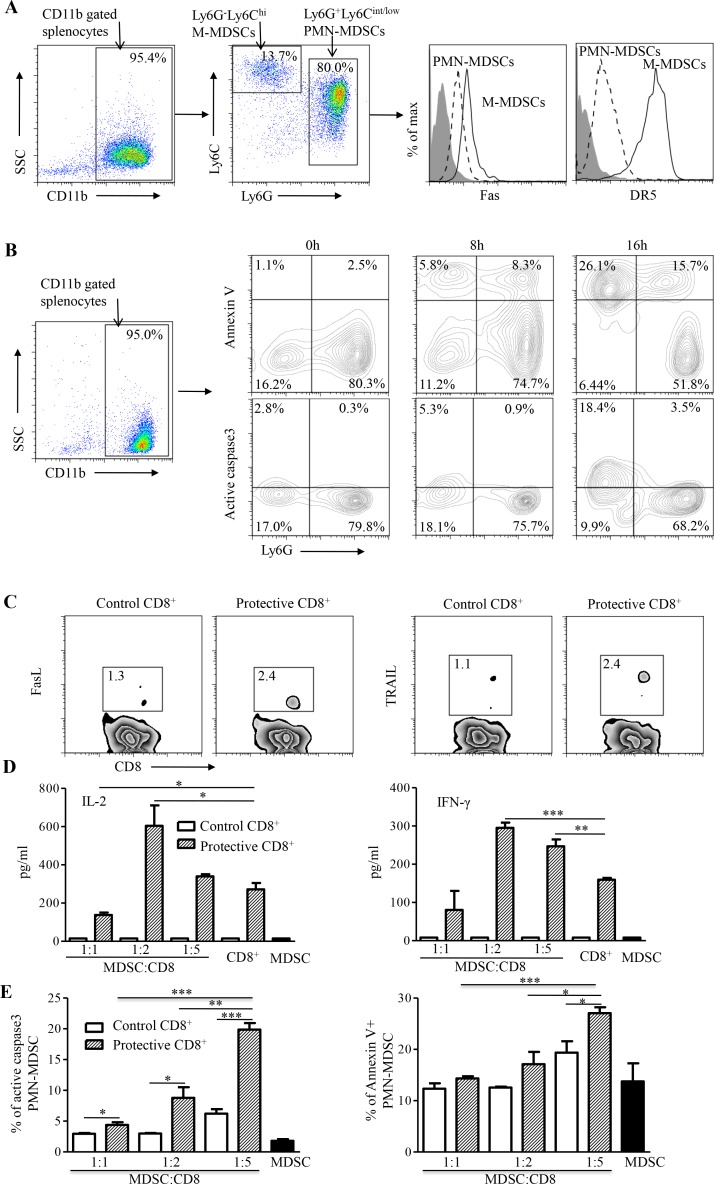
*In vitro* analysis of MDSCs and CD8^+^ T cells **A.** Flow cytometry analysis of MDSC subpopulations isolated from spleens of WT-AB1-bearing mice (left panel). Different levels of Fas and DR5 expression were found between PMN-MDSCs and M-MDSCs (right panel). **B.** The expression of active caspase-3 and annexin V overtime. The majority of M-MDSCs undergo apoptotic cell death faster than PMN-MDSCs during the *in vitro* culture. **C.** Flow cytometry analysis of FasL (left panel) and TRAIL (right panel) expression on CD8^+^ T cells isolated from spleens of group I and control mice. **D.** IL-2 and IFN-γ production from co-cultures of WT-AB1-induced MDSCs with group I CD8^+^ T cells but not with control CD8^+^ T cells at different MDSC:CD8^+^ ratios of 1:1, 1:2 and 1:5. Data represent mean ± s.e.m. Results are representative of three independent experiments. **E.** By measuring the expression of caspase-3 and annexin V, significant proportions of apoptotic PMN-MDSCs were detected in co-cultures with group I CD8^+^ T cells as compared with control CD8^+^ T cells at 24 h. Data represent mean ± s.e.m. Results are representative of three independent experiments. *P < 0.05; **P < 0.01; ***P < 0.001.

To investigate MDSC in a different experimental condition, we studied immuncompetent BALB/c mice. We previously demonstrated that the frequency of MDSCs can increase significantly in BALB/c mice, accounting for approximately 10% of total splenocytes after the transplantation of syngeneic AB1 mesothelioma [5]. Here, we found that the splenic MDSC population of AB1-bearing mice was consisted of two distinct subpopulations characterized by CD11b^+^Ly6G^+^Ly6C^low/int^ polymorphonuclear (PMN) MDSCs and CD11b^+^Ly6G^−^Ly6C^hi^ monocytic (M) MDSCs (Figure 6A). Moreover, PMN-MDSCs dominated over 80% of the total MDSCs in AB1-bearing mice. By measuring the expression of active caspase-3 and annexin V, we found that M-MDSCs were prone to apoptosis and had a significantly shorter life-span than PMN-MDSCs *in vitro* (Figure 6B). The higher levels of Fas and DR5 expression might render M-MDSCs more susceptible to apoptosis within 16 h *in vitro* (Figure 6A-6B) [23, 24].

We then sought to measure the interaction between CD8^+^ T cells and the dominant PMN-MDSCs. Protective CD8^+^ T cells and PMN-MDSCs purified from protected group I mice (Figure 3) and AB1-bearing mice, respectively, were co-cultured for analysis of active caspase-3 and annexin V expression on PMN-MDSCs by flow cytometry as described above (Figure 6B). As compared with control CD8^+^ T cells, protective CD8^+^ T cells not only exhibited relatively higher levels of TRAIL and FasL expression in two separate experiments (Figure 6C) but also released higher levels of IL-2 and IFN-γ (Figure 6D), and resulted in significant apoptosis of co-cultured PMN-MDSCs in a dose-dependent manner (Figure 6E). Interestingly, less IL-2 and IFN-γ were produced at MDSC:CD8 ratio of 1:1 as compared with CD8^+^ T cells alone (Figure 6D), indicating a likely suppressive effect of co-cultured CD8^+^ T cells by MDSCs. Conversely, when increased CD8^+^ T cells were presented at MDSC:CD8 ratios of 1:2 or 1:5 in the culture, significantly more IL-2 and IFN-γ were produced as compared with CD8^+^ T cells alone (Figure 6D). These data provide evidence that although MDSC may contribute to the suppression of CD8^+^ T cell function and support the progression of tumors [9], high frequency of functional tumor-specific PD1^−^Tim3^−^ CD8^+^T cells might eliminate PMN-MDSCs through apoptosis to counteract the suppressive effect of MDSCs while lead to impeded tumor growth [23, 24].

## DISCUSSION

A critical challenge in mesothelioma immunotherapy is to reveal the mechanism of efficacious vaccine responses. Here, we demonstrate that CD8^+^ T cells induced by the vaccine-mediated antigen spreading are tumor-specific and confer significant protection against the lethal challenge of wild-type mesothelioma. Besides the inverse correlation between the frequency of efficacious PD1^−^Tim3^−^ CD8^+^ T cells and that of MDSCs or tumor mass *in vivo*, these functional CD8^+^ T cells not only kill wild-type mesothelioma *in vivo* but also lead to apoptosis of the predominant PMN-MDSCs significantly in a dose-dependent manner. Our findings, therefore, may have implications to cancer immunotherapy.

Cancer immunotherapy has been hindered by poor CD8^+^ T cell responses against tumor-specific antigens because tumor cells often evolve to escape immune recognition and develop resistance through mutation or other mechanisms [25-27]. To avoid immune escape, one immunotherapy strategy is to target a broad repertoire of tumor antigens through vaccination of tumor cDNA library or combinatorial DNA vaccines [28, 29]. For the same purpose, another strategy is to provoke antigen spreading, a process that CD8^+^ T cell responses initiated by a vaccine trigger immune-mediated lysis of tumor cells, leading to the secondary immunity targeting at distinct and broader tumor antigens [30, 31]. The benefits of antigen spreading have been documented for vaccine-based immunotherapy against malignant melanoma and breast cancer [22, 32, 33]. Before the present study, however, it remained unknown if vaccine-mediated antigen spreading could be used for treating malignant mesothelioma. By a model vaccine-mediated antigen spreading, we show here that broad host immunity, including both humoral and cellular responses are elicited against wild-type mesothelioma. Moreover, we demonstrate that tumor-specific cytotoxic CD8^+^ T cells, but neither CD4^+^ T cells nor antibodies, confer the protection, suggesting that CD8^+^ T cells may play a central role in antigen spreading-mediated immunotherapeutic efficacy against malignant mesothelioma in mice.

In our previous study, using HIV-1 GAG-p24 in the model sPD1-p24_fc_/EP vaccine, we show that vaccine-elicited CD8^+^ T cells confer complete prevention and therapeutic cure of AB1-GAG malignant mesothelioma in immunocompetent BALB/c mice [5]. These efficacious CD8^+^ T cells display antigen-specific property and require a high frequency for function. Here, we show that the vaccine-mediated elimination of AB1-GAG mesothelioma results in the induction of tumor-specific CD8^+^ T cells through antigen spreading, which protect mice from the subsequent wild-type mesothelioma. sPD1-p24_fc_/EP vaccination achieved a functional state during the course of AB1-GAG regression (Figure 1-2) that not only prevents the rise of exhausted PD1^+^ and Tim3^+^ CD8^+^ T cells but also reduces tumor-induced MDSCs (Figure 5) [5]. It is possible that such a microenvironment is critical for the induction of high quality of tumor-specific effector PD1^−^Tim3^−^ CD8^+^T cells (Figure 5), which are capable of dramatically reducing the presence of MDSCs and improving immunotherapeutic responses in tumor-bearing mice [3]. However, it is still unknown which tumor antigens are responsible for eliciting the efficacious CD8^+^ T cells and therefore we are not able to measure their frequency and functionality. Nevertheless, our newly established experimental platform is useful for future identification of mesothelioma antigens responsible for the induction of protective effector CD8^+^ T cells. Since antigen-specific CD8^+^ T cells are enriched within mesothelioma [5], future investigation may unravel the protective tumor epitopes by focusing on these cells [34]. To this end, the detailed analysis of high avidity CD8^+^ T cell receptor in recognition of novel mesothelioma epitopes is warranted [35, 36]. Subsequently, these epitopes can be potentially tested in the context of PD1-based vaccine to amplify tumor-specific CD8^+^ T cells for therapeutic cure of mesothelioma [5].

Tumor-associated immunosuppressive network is a significant obstacle to an effective cancer immunotherapy [9]. Accordingly, regulatory T lymphocytes (Treg), tumor-associated MDSCs, tumor-associated macrophages, and dysfunctional dendritic cells may contribute to such a complex immunosuppressive network. Among them, MDSCs have emerged as one of the central regulators because they can inhibit T cell function potently and modulate the development of Treg cells [10, 12]. However, previous studies have shown that high frequency of broadly reactive anti-tumor T cell responses may lead to the reduction of MDSCs [5, 14]. Such reduction was thought of as a result of efficient elimination of tumor cells by efficacious CD8^+^ T cells, and therefore less MDSCs are induced by the regressing tumor. Few studies have demonstrated the elimination of tumor-induced MDSCs by efficacious CD8^+^ T cells. Here, we show that mesothelioma induces predominantly PMN-MDSCs *in vivo*. Moreover, PMN-MDSCs express death receptors Fas and DR5 (Figure 6A), making them prone to apoptosis by efficacious CD8^+^ T cells, which exhibit higher FasL and TRAIL expression and predominant IL-2/IFN-γ production (Figure 6C-6D) [23, 24]. Besides the significant inverse correlation between the frequency of functional PD1^−^Tim3^−^ CD8^+^T cells and that of MDSCs or tumor mass *in vivo* (Figure 5A-5B), in co-cultures with efficacious CD8^+^ T cells, a significant number of PMN-MDSCs underwent apoptosis in a dose-dependent way (Figure 6E). Thus, through initiation of MDSCs apoptosis to counteract their suppressive effect, higher frequency of efficacious CD8^+^ T cells could maintain their effector function (Figure 6C) [37]. This new finding indicates that vaccine-induced CD8^+^ T cells capable of eliminating both tumor cells and MDSCs are likely necessary for an effective immunotherapy against the malignant mesothelioma [23, 24].

## MATERIALS AND METHODS

### Animal and cell lines

All animal experiments were approved by the Committee on the Use of Live Animals in Teaching and Research (CULATR) of The University of Hong Kong (HKU, #2438-11). 5- to 8-week-old female BALB/c and C.B-17/Icr-scid (SCID) mice were maintained according to standard operational procedures at HKU Laboratory Animal Unit (LAU). AB1 cells were purchased from European Collection of Cell Cultures and transduced with a lentiviral vector to express luciferase in WT-AB1 or luciferase together with HIV-1 GAG in AB1-GAG as previously described [5]. Transduced WT-AB1 and AB1-GAG were maintained in RPMI-1640 medium supplemented with 10% FBS and 1μg/ml puromycin (Invitrogen).

### Mouse immunization and tumor challenge

Groups of BALB/c mice were intramuscularly (i.m.) immunized with 100 μg of plasmid DNA of sPD1-p24_fc_, p24_fc_ or PBS control in a total volume of 100 μl, followed immediately by electroporation (EP) over the injection site using the TERESA gene delivery device (Shanghai Teresa Bio-Tech Co., Ltd.) as previously described [6]. The DNA/EP vaccination was repeated twice at three-week intervals. For tumor challenge, 5 × 10^5^ AB1-GAG cells were inoculated in the left flank subcutaneously (s.c.). Tumor implantation was repeated two more times at two-week intervals. The final tumor challenge was performed s.c. with a lethal dose of 5 × 10^5^ WT-AB1 cells in the right flank. *In vivo* bioluminescence imaging (BLI) was taken once a week during the experiment and the luciferase intensity was measured in the region of interest (ROI) using the IVIS100 Imaging System (Caliper Life Science). Tumor size was also measured using calipers. Based on the CULATR guidelines, mice were sacrificed when tumors reached a size greater than 15 mm.

### Splenocyte collection and T cells isolation

To wash the cells out of the spleen capsule, a tuberculin syringe containing PBS was used to inject the spleen in several places. Splenocytes were collected for T cell isolation using the mouse splenocyte separation medium (Dakewei biotech) via the discontinuous gradient centrifugation. CD4^+^ or CD8^+^ T cells were then purified using Dynabeads Untouched T Cell Kits according to the manufacturer's instruction (Invitrogen).

### *In vitro* T cell cytotoxicity assay

Luciferase-expressing WT-AB1 cells (2 × 10^4^/well) were cultured in 96-well plate one day before the addition of purified CD8^+^/CD4^+^ T cells at E/T ratios from 0.5:1 to 20:1. Bioluminescence imaging (BLI) was taken 6 hours after incubation. The level of luminescence activity reflects the degree of CTL-mediated killing of tumor cells under the IVIS100 Imaging System (Caliper Life Science) as previously described [38].

### Antibody-dependent cell mediated cytotoxicity (ADCC) assay

The LIVE/DEAD Viability/Cytotoxicity Kit (Invitrogen) was used in this assay. Briefly, WT-AB1 cells (2 × 10^4^/well) were resuspended in 1:250 diluted 3, 3′-dioctadecyloxacarbocyanine (DiO) solution and incubated for 20 min in the 37°C tissue culture incubator. WT-AB1 cells were then washed and resuspended in 1:100 or 1:1000 diluted anti-sera, respectively. Effector splenocytes (1 × 10^7^/ml and 100 μl) were transferred into each tube to mix with target WT1-AB1 cells at an effector-to-target (E/T) ratio of 50:1. Mixed cells were incubated for 4 hours in the 37°C incubator. After that, cells were collected and transferred into fresh culture medium containing 1:500 diluted propidium iodide (PI) and incubated for 10 min before analysis on a FACSCalibur instrument (BD Bioscience) and Flowjo software (Tree Star, version 7.6).

### Adoptive transfer of T cells

SCID mice were inoculated s.c. with 5 × 10^5^ WT-AB1 cells on their left flank. Tumors were left to grow for 7 days until they were visible (approximately 5 mm in length). Cyclophosphamide (Cy) was injected intraperitoneally (150 mg/kg) one day before the adoptive transfer to promote the activation and proliferation of transferred cells without affecting tumor growth [39]. On the following day, 2 × 10^6^ of isolated CD8^+^ T cells in 100 μl PBS were injected *via* tail vein into each mouse.

### MDSC isolation and culture

MDSCs from AB1-bearing BALB/c mice were isolated from their spleens using the Myeloid-Derived Suppressor Cell Isolation Kit according to the manufacturer's protocols (Miltenyi Biotech). The purity of PMN-MDSCs (CD11b^+^Ly6G^+^Ly6C^low/int^) and M-MDSCs (CD11b^+^Ly6G^−^Ly6C^hi^) was greater than 95% as determined by flow cytometry. To assess CD8^+^ T cells mediated apoptosis, MDSCs and freshly isolated CD8^+^ T cells were co-cultured at MDSC-to-CD8^+^ T-cell ratios of 1:1, 1:2, and 1:5 in the 37°C tissue culture incubator for 24 hours before they were subjected to analysis of active caspase-3 and annexin V expression. Cell-free supernatants were also collected from the co-culture for cytokine quantification using the mouse Th1/Th2/Th17 10-plex FlowCytomix Kit according to the manufacturer's instruction (eBioscience).

### Flow cytometry

Monoclonal antibodies (mAbs) specific for mouse cell surface markers CD3, CD4, CD8, CD11b, Gr-1, Ly6G, Ly6C, Fas and DR5 were all purchased from eBiosciences. The intracellular staining of cleaved caspase-3 was performed using BD Cytofix/Cytoperm kit. The mAbs specific for the cleaved caspase-3 and Annexin V were obtained from BD Bioscience. The staining protocols were followed according to the manufacturer's recommendations (BD Biosciences).

### Statistical analysis

A two-tailed Student t-test was performed to determine statistical significance between different groups. P value less than 0.05 was considered to be statistically significant. Survival of mice was plotted to a Kaplan-Meier survival curve and the observed difference was determined by the log-rank test (GraphPad Prism 5 software). Data are presented as the mean values ± standard error (s.e.m.).
